# Comparison of Visual Evoked Potentials and Retinal Nerve Fiber Layer Thickness in Alzheimer’s Disease

**DOI:** 10.3389/fneur.2013.00203

**Published:** 2013-12-16

**Authors:** Robert Kromer, Nermin Serbecic, Lucrezia Hausner, Lutz Froelich, Sven C. Beutelspacher

**Affiliations:** ^1^Department of Ophthalmology, Medical Faculty Mannheim, Ruprecht-Karls-University Heidelberg, Mannheim, Germany; ^2^Division of Geriatric Psychiatry, Central Institute of Mental Health, Medical Faculty Mannheim, Ruprecht-Karls-University Heidelberg, Mannheim, Germany

**Keywords:** Alzheimer’s disease, dementia, retinal nerve fiber layer thickness, visual evoked potentials, optical coherence tomography

## Abstract

**Introduction:** Alzheimer’s disease (AD) is a long term progressive neurodegenerative disease and might affect the retinal nerve fiber layer thickness (RNFLT) of the eye. There is increasing evidence that visual evoked potentials (VEP), which are an objective way to indicate visual field loss, might be affected by the disease as well.

**Materials and Methods:** About 22 patients (mean age: 75.9 ± 6.1 years; 14 women) with mild-to-moderate AD and 22 sex-matched healthy patients were examined. We compared the use of VEP and RNFLT using the latest high-resolution spectral domain optical coherence tomography with eye-tracking capabilities for optimized peripapillary scan centering for the first time in AD patients.

**Results:** The mean MMSE score was 22.59 ± 5.47 in the AD group, and did not significantly correlate with the VEP latencies. We found no significant difference between the VEP latencies of the AD patients and those of the control patients. No peripapillary sector of the retina had a RNFLT significantly correlated with the VEP latencies.

**Discussion:** We demonstrated that pattern VEP did not show any significant correlation despite subtle loss in RNFLT. It remains open whether additional flash VEP combined with RNFLT analysis may be useful in diagnosing AD, particularly for mild-to-moderate stages of the disease.

## Introduction

Alzheimer’s disease (AD) is described as a long term progressive neurodegenerative disease and characterized by a large inter-subject variability. The pathogenesis of AD is still an open debate. In most cases, the clinical diagnosis of AD is made early enough and is accurate, however paraclinical support would be useful for all cases, and even more so if the disease course, namely neurodegenerative changes and therapeutic effects, could be monitored and objectified over time. Drugs which have been used for the therapy of AD might be most effective in the early stages of the disease (1). Traditional neuro-imaging diagnostics including magnetic resonance imaging (MRI) to show the atrophy of the medial temporal lobe (2, 3), positron emission tomography (PET) to detect changes in the metabolism of glucose (3, 4) and the Aβ storage (5, 6), and the cerebrospinal fluid analysis (CSA) to measure the biomarkers for tau protein and Aβ levels (7, 8) have only limited value (9).

A new ophthalmic imaging method called optical coherence tomography (OCT) has been considered to be useful for the detection of early stages of AD (10–12). OCT provides cross-section imaging of the retina and is able to measure the retinal nerve fiber layer thickness (RNFLT) (13, 14). It has been used for the diagnosis of many diseases of the retina and various optic neuropathies, including glaucoma (15–22). The human eye is an embryological protrusion of the brain, and the nerves and axons of the retinal nerve fiber layer are similar to those of the brain. This leads to the question of whether AD might also affect the RNFLT of the eye. For many years histopathological studies have shown that the RNFLT is not affected by AD (23, 24). In contrast to this, recent OCT studies, claimed to be able to detect a loss of the RNFLT in patients with AD (11, 12, 25–27). However, those studies were performed using conventional OCT technology, lacking reliable optic nerve scan centering (21), a subject to potential misinterpretation in a number of cases (21). Studies showed that OCT measurements of the RNFLT were associated with mild cognitive impairment (MCI), and this could be taken to discriminate between different stages of AD (10–12).

Visual evoked potentials (VEP) are an objective way to indicate visual field loss (28–31). They represent the visual cortex activity, as they are a reaction to the visual information that is passing through the optical media of the eye which gets processed by the retina and the genicula-striate pathway. The occurring evoked response shows the performance of the visual system until that point. There are numerous studies discussing the use of VEP in AD. Some studies have shown that AD patients have longer latencies of flash VEP (32–34). Contrary to this, other studies showed normal pattern VEP while the RNFLT, as measured with conventional OCT technology, presented abnormal in AD patients (11).

The purpose of this study was to compare the use of VEP and RNFLT using the latest high-resolution spectral domain optical coherence tomography (SD-OCT) with eye-tracking capabilities for optimized peripapillary scan centering for the first time in AD patients. The goals were to show (i) whether there is any change of VEP in AD patients as compared to sex-matched controls, and (ii) to correlate those latencies with the RNFLT of the AD patients.

## Materials and Methods

About 42 eyes of 22 patients (mean age: 75.9 ± 6.1 years; 14 women) with mild-to-moderate AD were examined – two patients had only one eye examined. The control group involved 43 eyes of 22 sex-matched healthy patients (mean age: 64.0 ± 8.2 years; 15 women) – one patient had only one eye examined. The study was approved by the Ethics committee II of the Medical Faculty Mannheim, Ruprecht-Karls-University Heidelberg, Mannheim, Germany and followed the ethical principles of the Declaration of Helsinki. Before the examinations, informed consent was obtained from each patient. In case the patient was incompetent of giving informed consent, the care giver (spouse or adult child) declared their approval.

Alzheimer’s disease was diagnosed by a physician and a multi-professional team (neurologists, psychiatrists, psychologists) at the Memory Clinic of the Department of Geriatric Psychiatry of the Central Institute of Mental Health, Mannheim, Germany. The assessments consisted of the medical history from the patient and caregiver, general exams, laboratory, neuropsychological, and radiologic tests. To indicate progressive cognitive decline of everyday functioning and to show the absence of any neurological or psychiatric disorder possibly causing dementia (other than AD) general physical, neurological, and neuropsychological examinations were performed. The laboratory testing included complete blood count with differential counts, syphilis screening, serum electrolytes, liver and renal function, cholesterol status, thyroid function, serum vitamin B12, and folate levels. This way secondary causes of dementia were excluded. The neuropsychological tests (CERAD test battery WMS-LM, TMT-A and -B) were used to show an impairment in one or two more clinical domains severe enough to cause impairment in activities of daily life. For radiologic testing MRI was used to show a visual rating of evidence of medial temporal lobe atrophy and absence of major white matter abnormalities and/or other cerebrovascular disorders. In addition to that most patients had lowered Aβ 42 peptide and accelerated tau and/or phospho-tau in cerebrospinal fluid (which is typical for AD).

The patients’ history and medical records were carefully reviewed for diseases possibly affecting the RNFLT. Only patients meeting inclusion and exclusion criteria were included. The ophthalmic inclusion criteria were (i) best-corrected visual acuity of 0.3 LogMAR or better, (ii) spherical refraction within ±5.0 diopters (D), (iii) cylindrical correction within ±2.0 D, and (iv) normal results for visual field testing (Swedish Interactive Thresholding Algorithm SITA; Octopus 101 Perimeter; Haag-Streit Deutschland GmbH, Wedel, Germany). The exclusion criteria were (i) intensive alcohol abuse, (ii) body mass index >30, (iii) intraocular pressure ≥21 mmHg, (iv) history of glaucoma, (v) anterior ischemic optic neuropathy, (vi) high myopia, (vii) prior ocular surgery, and (viii) congenital abnormal abnormalities of the optic nerve.

Patients underwent various ophthalmic examinations: (i) assessment of best-corrected visual acuity by auto-refractometry (OCULUS/NIDEK auto-refractometer, OCULUS Optikgeräte GmbH, Wetzlar, Germany) followed by subjective refractometry using the ETDRS (Early Treatment of Diabetic Retinopathy Study) 2000 chart for high-contrast visual acuity, (ii) slit lamp assisted biomicroscopy of the anterior segment, (iii) ophthalmoscopy after medical dilation of the pupil, (iv) visual field testing (Swedish Interactive Thresholding Algorithm SITA; Octopus 101 Perimeter; Haag-Streit Deutschland GmbH, Wedel, Germany), (v) Goldmann applanation tonometry, (vi) VEP measurement, (vii) OCT for RNFLT measurement.

The VEP were carried out with ROLAND CONSULT RETI-port/scan 21 (ROLAND CONSULT, Brandenburg an der Havel, Deutschland) in abidance of the current guideline for pattern VEP of the International Society for Clinical Electrophysiology of Vision (35). The pattern-reversal protocol used black and white checks changing phase abruptly and repeatedly at a reversal rate of two reversals per second (±10%) (Each full cycle consists of two reversals so this equates a frequency of 1.0 Hz.). The checkerboard stimuli with large 1° (60 min of arc) and small 0.25° (15 min) checks provided N75 and P100 peaks, where P100 peaks are the most prominent ones.

The thickness of the RNFLT was measured using latest high-resolution SD-OCT (SPECTRALIS software version 5.3.3.0, EYE EXPLORER Software 1.6.4.0; Heidelberg Engineering, Heidelberg, Germany). This device is a combination of normal SD-OCT and confocal scanning laser ophthalmoscopy (cSLO). The superluminescence diode of the SD-OCT emits a scan beam with a wavelength of 879 nm performing up to 40,000 A-scans/s with a depth resolution of 7 μm and a transversal resolution of 14 μm. The cSLO scans point to point with a laser the illuminated retina and creates this way a real-time reference fundus image. SD-OCT examinations are fast, inexpensive, objective, and non-dependent on the cooperation of patient. The SD-OCT has several additional features that were used in this study: eye-tracking (TrueTrack™; Heidelberg Engineering, Heidelberg, Germany) locks the SD-OCT image to the reference fundus capture and enables comparing repeated measurements. Furthermore, the examination is non-dependent on the investigator due to the enabled eye-tracking. The automatic real-time averaging mode (ART) takes additional B scans in order to improve the quality. Therefore, the device locks after the first A scan and adds B scans whenever the eye has the exact same position. These two features combined with the high speed of the device facilitate reducing artifacts. In our study at least three high-resolution images with enabled ART mode (with at least 18 additional frames) were taken. Scans with low fixation or failing RNFL segmentation were excluded. To minimize possible variabilities, all images were performed by one examiner. The criteria for determining the scan quality were: (i) a clear fundus image before and after the acquisition; (ii) absence of scan or algorithm failures; (iii) even and dense gray scale saturation in all retinal layers and dense gray visible in retinal pigment epithelium; and (iv) RNFL visible as a continuous scan pattern.

Statistical analysis was carried out using a commercially available software package (Prism 6 for Mac OSX; GraphPad Software, Version 6.0b). Mean and standard deviations were presented. *P*-values were corrected according to Bonferroni to correct for performing multiple statistical analyses. All *P*-values were two-tailed and a *P*-value <0.05 was considered to indicate statistical significance. Correlation was done using Pearson correlation calculations as values are sampled from populations that follow a Gaussian distribution – at least approximately. By correlation coefficient *r* is meant. VEP comparison and RNFLT are presented as Tukey box-and-whiskers plots with values that are above or below the whiskers drawn as individual dots. The mean of both eyes of each participant was used for statistical analysis (phenotype). VEP normative data standardization was established following the guidelines of the International Society for Clinical Electrophysiology of Vision (35), resulting in a 95% reference interval as the limit of normal (range from 2.5 to 97.5%).

## Results

**Figure 1:**
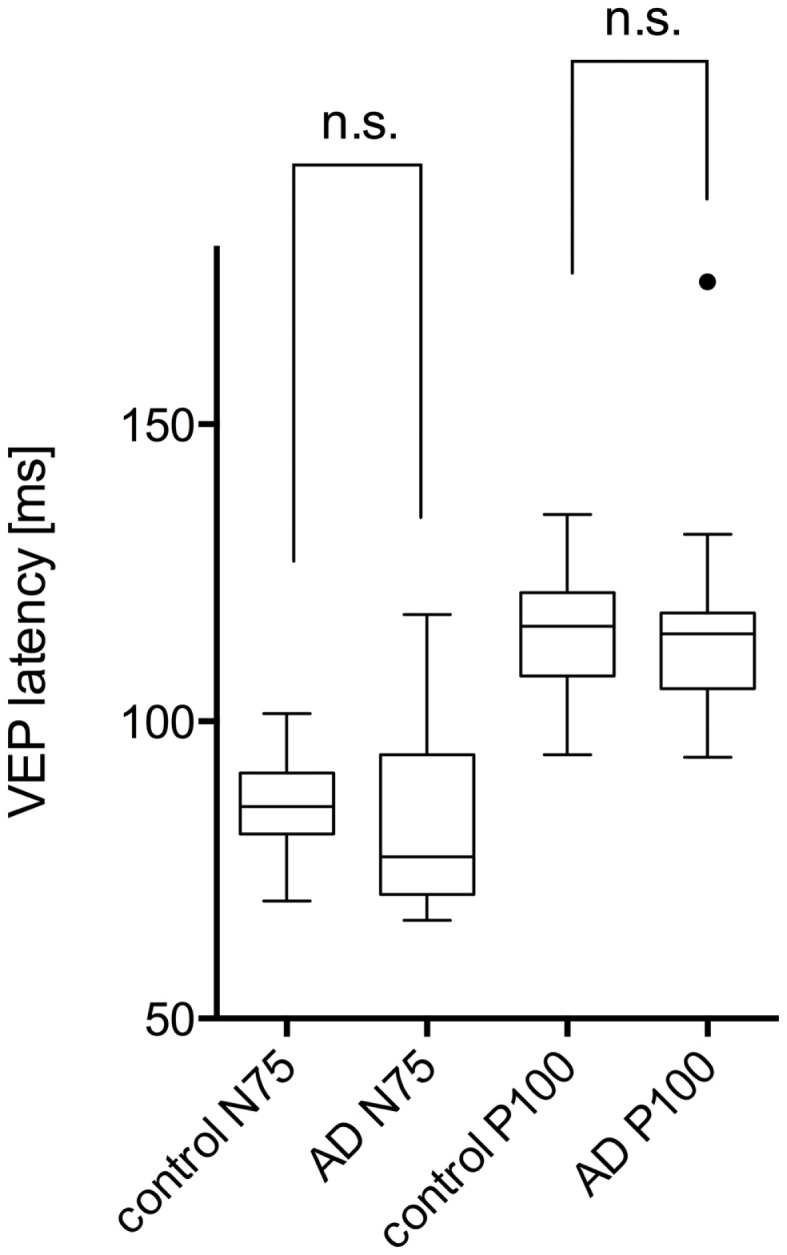
**Comparison of VEP latencies (N75 and P100) of control patients (marked as control) and AD patients (marked as AD)**. No significant (marked as n.s.) differences were found between the two groups (*P*-value >0.05). Data is presented as a Tukey box-and-whiskers plot, with values that are above or below the whiskers drawn as individual dots.

**Figure 2:**
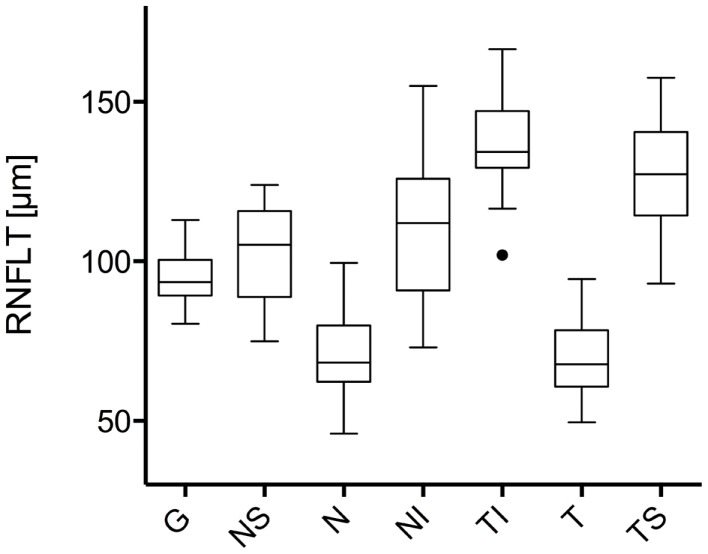
**Comparison of the retinal nerve fiber layer thickness in different peripapillary sectors: the different sectors are labeled as follows: global (G), nasal superior (NS), nasal (N), nasal inferior (NI), temporal inferior (TI), temporal (T), and temporal superior (TS)**. Data is presented as a Tukey box-and-whiskers plot, with values that are above or below the whiskers drawn as individual dots.

**Figure 3:**
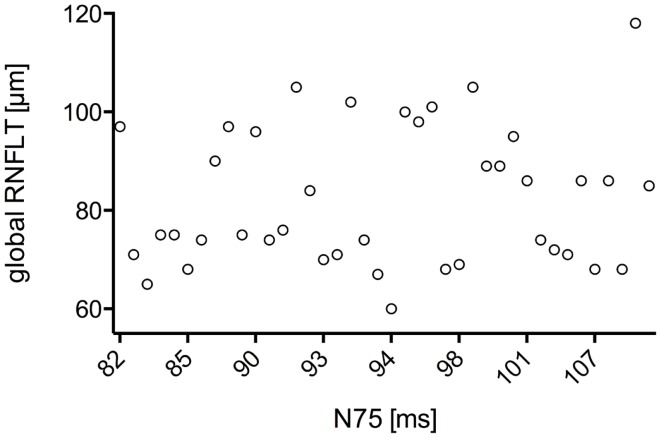
**Scatter plot of all N75 latencies in dependence of the associated global RNFLT**. There was no significant correlation found for N75 latency with global RNFLT in AD patients (*r* = 0.271;*P*-value = 0.222).

**Figure 4:**
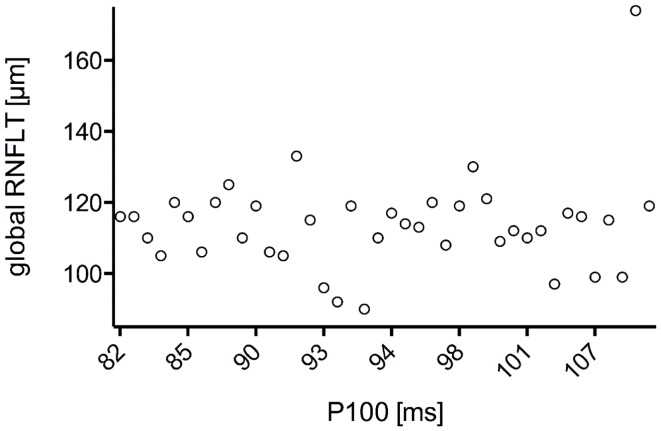
**Scatter plot of all P100 latencies in dependence of the associated global RNFLT**. There was no significant correlation found for N75 latency with global RNFLT in AD patients (*r* = 0.322;*P*-value = 0.144).

The study included 42 eyes of 22 patients with AD (mean age: 75.9 ± 6.1 years; age range: 66–88 years; 14 women), and 43 eyes of 22 sex-matched healthy control patients (mean age: 64.0 ± 8.2 years; age range: 53–85 years; 16 women). In both groups, there was no significant correlation found between male or female and the right or left eye in RNFLT and VEP latencies (*P*-value >0.05). There was no significant correlation of VEP latencies and RNFLT with the age of the participants in both groups. The mean MMSE score was 22.59 ± 5.47 in the AD group, and this was not significantly correlated with the VEP latencies. We found no significant difference between the VEP latencies of the AD patients and those of the control patients (*P*-value >0.05), as presented in Figure 1. Furthermore, all AD patients had normal VEP values within the 95% reference interval as the limit of normal (range from 2.5 to 97.5%). The RNFLT of the AD patients is presented in Figure 2. Using our previously introduced advanced gender-matched measurement model, the RNFLT was marked as pathologic in 32 out of 42 eyes (76.19%) (36). In order to show the relationship between VEP latencies and RNFLT of the AD patients globally, we outlined two scatter plots (Figures 3 and 4). No correlation was found between these variables. In more detail, no peripapillary sector of the retina had a RNFLT that significantly correlated with the VEP latencies (Table 1; *P*-value >0.05).

**Table 1:** **Correlation of N75 and P100 latencies with the RNFLT of the different sectors**.

	G	NS	N	NI	TI	T	TS
N75	0.271 *P* = 0.222	0.363 *P* = 0.096	0.344 *P* = 0.117	−0.027 *P* = 0.905	0.091 *P* = 0.689	0.068 *P* = 0.763	0.139 *P* = 0.537
P100	0.322 *P* = 0.144	0.194 *P* = 0.386	0.359 *P* = 0.101	0.261 *P* = 0.242	0.155 *P* = 0.491	0.015 *P* = 0.946	0.145 *P* = 0.519

*The different sectors are labeled as follows: global (G), nasal superior (NS), nasal (N), nasal inferior (NI), temporal inferior (TI), temporal (T), and temporal superior (TS). The correlation tests with Bonferroni correction for multiple comparisons showed no significant correlations. The table shows the correlation coefficients and the *P*-value below. A *P*-value <0.05 is considered significant (with Bonferroni correction for multiple comparison in this case a *P*-value <0.0036 is considered significant)*.

## Discussion

Our results showed normal pattern VEP latencies compared to a sex-matched healthy control group. Furthermore, the RNFLT of the AD patients did not correlate with pattern VEP latencies.

The use of VEP in AD has been discussed a lot in recent years. Some studies found abnormal pattern VEP in AD patients (33, 37, 38). In contrast, a number of authors stated that they did not find any abnormalities in pattern VEP while experiencing abnormal flash VEP (39–43). Nevertheless, flash VEP suffer from a lower reproducibility compared to pattern VEP (35). One study comparing RNFLT and VEP in AD found normal pattern VEP with abnormal RNFLT, however using conventional OCT technology (11). The RNFLT has been examined in several studies using histopathologic techniques, with inconsistent findings. Some stated that there is no decrease (44–48), while others found abnormal RNFLT (23, 24). Additionally, studies using conventional time-domain OCT technology, lacking eye-tracking capabilities, found only a subtle RNFLT decrease in AD patients (10–12, 25, 49–51).

The different findings between flash and pattern VEP may result from the different methodological approach. The functional organization of the proximal retina and the visual cortex is preferentially sensitive to patterned stimuli (52). As the changes in RNFLT are mostly subtle and/or subclinical, there may be no effect in pattern VEP while experiencing abnormal flash VEP. The disagreement in OCT studies may also evolve from divergent stages of the disease and the technical inaccuracy of post-mortem histopathological studies. In our case all AD patients had a mild-to-moderate AD.

Our results implicate that pattern VEP does not show any changes in AD despite the fact that RNFLT changes demonstrated as a decrease in one or more peripapillary sectors, were found in some but not all AD patients. As a consequence, pattern VEP did not correlate with the RNFLT of AD patients. However, severe stages of the disease may show further changes. Nevertheless, literature indicates that the use of flash VEP may be recommended to show changes in AD.

The limitations of this study were, (i) that no flash VEP was used to show the difference to pattern VEP, (ii) that only cross-sectional measurements were done without follow-up to investigate a potential progression of the RNFLT and VEP, and (iii) that the patients had a mild-to-moderate AD. On the other hand, the strengths were that (i) patients and controls were sex-matched, (ii) for the first time latest high-resolution SD-OCT measurements were obtained, and (iii) the clinical diagnosis of AD was obtained in a specialized memory clinic by using highly standardized clinical criteria, including neuropsychological assessments and biomarkers (MTA, CSF).

In summary, we demonstrated that pattern VEP may not be useful for diagnosing AD and may not show a correlation despite subtle loss in RNFLT. It remains unclear whether flash VEP combined with RNFLT analysis may be useful in diagnosing AD, taken in account that flash VEP have a lower reproducibility compared to pattern VEP. For further studies, it is important for the interpretation of SD-OCT- and VEP-investigations that the standardized technical requirements provided by latest SD-OCT technology are used and the current guidelines are applied.

## Conflict of Interest Statement

The authors declare that the research was conducted in the absence of any commercial or financial relationships that could be construed as a potential conflict of interest.
